# Genetic variations at 8q24 and gastric cancer susceptibility: A meta-analysis study

**DOI:** 10.1371/journal.pone.0188774

**Published:** 2017-12-12

**Authors:** Pengke Zhi, Jian Shi, Feng Liu

**Affiliations:** 1 Henan Key Laboratory of Cancer Epigenetics, Cancer Institute, The First Affiliated Hospital and College of Clinical Medicine of Henan University of Science and Technology, Luoyang, Henan, China; 2 Department of Neurology, The First Affiliated Hospital Of Henan University Of Science and Technology, Luoyang, Henan, China; 3 Department of Gastroenterology and Hepatology, Guangzhou First People’s Hospital, Guangzhou Medical University, Guangzhou Guangdong, China; National Cancer Center, JAPAN

## Abstract

**Background:**

Published data on the association between genetic variants on the 8q24 chromosome and gastric cancer (GC) susceptibility are inconclusive. Here we present a meta-analysis designed to evaluate the relationship between 8q24 variants (single nucleotide polymorphisms (SNPs) labeled rs6983267 and rs1447295) and risk of developing GC.

**Methods:**

A literature search was performed using studies published on PubMed, Science Direct, OVID and Web of Science databases up to December 2016. Studies were selected based on our enrollment criteria, relevant data was extracted from each study and the odds ratios (OR), and 95% confidence intervals (CI) were calculated and used to assess the strength of associations found between 8q24 polymorphisms and GC risk. Conclusions about acceptable strong associations were made after taking into account sample heterogeneity and sensitivity analyses.

**Results:**

A total of seven studies containing ten case-control studies were selected. Among these studies were six studies of 1,421 GC patients and 3,393 controls examining the role of the rs6983267 SNP and four studies including 779 cases and 1,266 controls examining rs1447295 SNP. The pooled results of these studies indicated that there was no significant association between both genetic variants and GC susceptibility using an allele, dominant, recessive and homozygote genetic models. When using a heterozygote genetic model, a significant increase was found in the association of GC risk for rs6983267 SNP (OR = 1.07, 95% CI = 1.01–1.12, *P* = 0.015), whereas for rs1447295 SNP a significant decreased risk was detected (OR = 0.82, 95% CI = 0.69–0.98, *P* = 0.030). In subgroup analyses based on ethnicity and genotyping methods, similar non-significant results were observed for the rs1447295 variant using the four genetic models (allele, dominant, recessive or homozygote models) and for the rs6983267 variant using only the allele, recessive and homozygote models. However, after a multiple testing correction to our calculations, these associations remained non-significant.

**Conclusion:**

Meta-analysis of gastrointestinal cancer genetic analysis studies did not confirm an association between 8q24 chromosome polymorphisms (specifically rs6983267 and rs1447295) and susceptibility to GC in the general populations.

## Introduction

Gastric cancer (GC), the fifth most common cancer worldwide, is rampant in many countries around the world, causing more than 841,000 deaths each year [1]. Like all solid tumors, GC is understood to be the result of the interactions of environmental and genetic factors. It has been suggested that environmental risk factors, including *helicobacter pylori* (*H*. *pylori*) infection [2], alcohol consumption [3], cigarette smoking [4] and a high-salt diet [5], may be involved in the GC pathogenesis. However, it has also been shown that among people who are exposed to the same environmental carcinogenic factors, only a fraction develop GC. This finding suggests that there are discrepancies in the genetic background of susceptible people which may lead to individual differences in GC susceptibility [6, 7].

Large genome-wide association studies (GWAS) have revealed that various genetic variants on chromosome 8q24 are associated with an increased risk for multiple cancer types (e.g., ovary [8], breast [9], colorectal [10], prostate [11]). Interestingly, within the 8q24 locus lies a 1.18 Mb region that contains unknown genes. Downstream of this so called “gene desert” are two candidate cancer susceptibility genes, the *OCT4* pseudogenes *POU5F1P1* and *c-MYC*. Over the last two decades, a number of molecular epidemiological studies have investigated the link between two polymorphisms, rs6983267 and rs1447295, in this region and analysis of association to GC susceptibility have revealed inconsistent results. Several studies suggest that the rs6983267 polymorphism is associated with an increased risk of GC [12, 13], while an inverse association was seen with rs1447295 polymorphisms [14]; however other studies have failed to confirm such associations [15–18]. Taking into account all of these controversial results, we executed the first meta-analysis study to evaluate the existence of any association between polymorphism variants on the 8q24 chromosome (specifically rs6983267 and rs1447295) and GC susceptibility.

## Materials and methods

### Identification of relevant studies

We performed a systematic literature search in four databases: PubMed, Science Direct, OVID and Web of Science for studies evaluating the relation between 8q24 locus polymorphisms (rs6983267 and rs1447295) and GC susceptibility, published up to December 15, 2016. Various combinations of search terms were used to search for our genetic location of interest (“8q24”, “rs1447295” or “rs6983267”) and gastric cancer (“gastric cancer”, “gastric carcinoma”, “gastric tumor”, “gastric neoplasm”, “stomach cancer”, “stomach carcinoma”, “stomach tumor”, “stomach neoplasm”) and genetic variant (“polymorphism”, “variation”, “allele” or “genotype”); these terms were used in the search process without language restriction. Once suitable studies were selected from the search results, the reference sections of these articles were also carefully screened for other data relevant to our meta-analysis.

### Study selection

A study was selected for enrollment in this meta-analysis if it satisfied all of the following criteria: 1) studies evaluating the association between rs6983267/rs1447295 variant and GC; 2) original case-control or cohort designed studies in humans; 3) studies with sufficient genotype distribution data for calculation of combined odds ratios (OR) and 95% confidence intervals (CI). When researches did not report detailed information regarding the genotype distribution in each group, the corresponding authors of the study were contacted for unpublished data. If the same data were shared in more than one study, only the study with the largest sample size was included to avoid duplication. The quality of enrolled studies was assessed using the Newcastle-Ottawa Scale (NOS) [19], which used three main selection factors (selection, comparability and exposure). This study gives one star for each satisfactory answer. Studies with stars equal to or higher than five were considered to be of high quality.

### Data extraction

Three investigators separately extracted the available data from each included study. The extracted data were as following: first author, year of publication, study design, number of cases and controls, study of population characteristics (country, ethnicity, mean age or gender), *H*. *pylori* infection, source of control, genotyping method, genotype distribution in cases and controls. To ensure the accuracy of extracted information, each of the three investigators cross-checked their extraction results with each other; any discrepancies were discussed until a consensus about valid data was reached.

### Statistical analysis

Stata version 10 software (Stata Corporation, College Station, TX, USA) were applied to carry out statistical analysis. To measure strength of association, the summary OR and 95% CI were calculated for each genetic variant using the random-effect model (DerSimonian-Laird method) [20] or the fixed-effect model (Mantel-Haenszel method) [21]. The significance of the summary OR was confirmed using a Z-test. A *P* value < 0.05 was considered to be statistically significant. In order to refrain from excessive comparisons, five genetic models were employed to compute global data: an allele model (guanine (G) vs. thymine (T) alleles for rs6983267 and adenine (A) vs. cytosine (C) alleles for rs1447295), a dominant model (GG+GT vs. TT for rs6983267 as well as CA+AA vs. CC for rs1447295), a recessive model (GG vs. GT+TT for rs6983267 and AA vs. CC+CA for rs1447295), a homozygote model (GG vs. TT rs6983267 as well as AA vs. CC for rs1447295) or a heterozygote model (GT vs. TT for rs6983267 and CA vs. CC for rs1447295). Because of the need for multiple comparisons within our dataset, our meta-analysis was performed 50 times. The Bonferroni correction was employed to adjust for the multiple testing of our dataset. A *P* value less than 0.05/50 (0.001) was accepted as statistically significant after a Bonferroni correction. In order to test the heterogeneity of the dataset, statistical analysis using a Q-test and Higgins *I*^*2*^ statistic were conducted. If a large amount of heterogeneity was found (*I*^*2*^ greater than 50%) then a random-effect model was adopted, otherwise a fixed-effect model was used [22]. Subgroup analyses were performed to explore possible sources of heterogeneity by examining differences of study subject ethnicity (Asian vs. non-Asian) and genotyping method used to process samples (PCR-RELP/TaqMan vs. other). A sensitivity analysis was completed to justify the reliability of our conclusions. Finally, an Egger’s test was used to assess the publication bias of included studies [23]. Publication bias was thought to be significant when the *P* value was less than 0.1.

## Results

### Study characteristics

**Fig 1:**
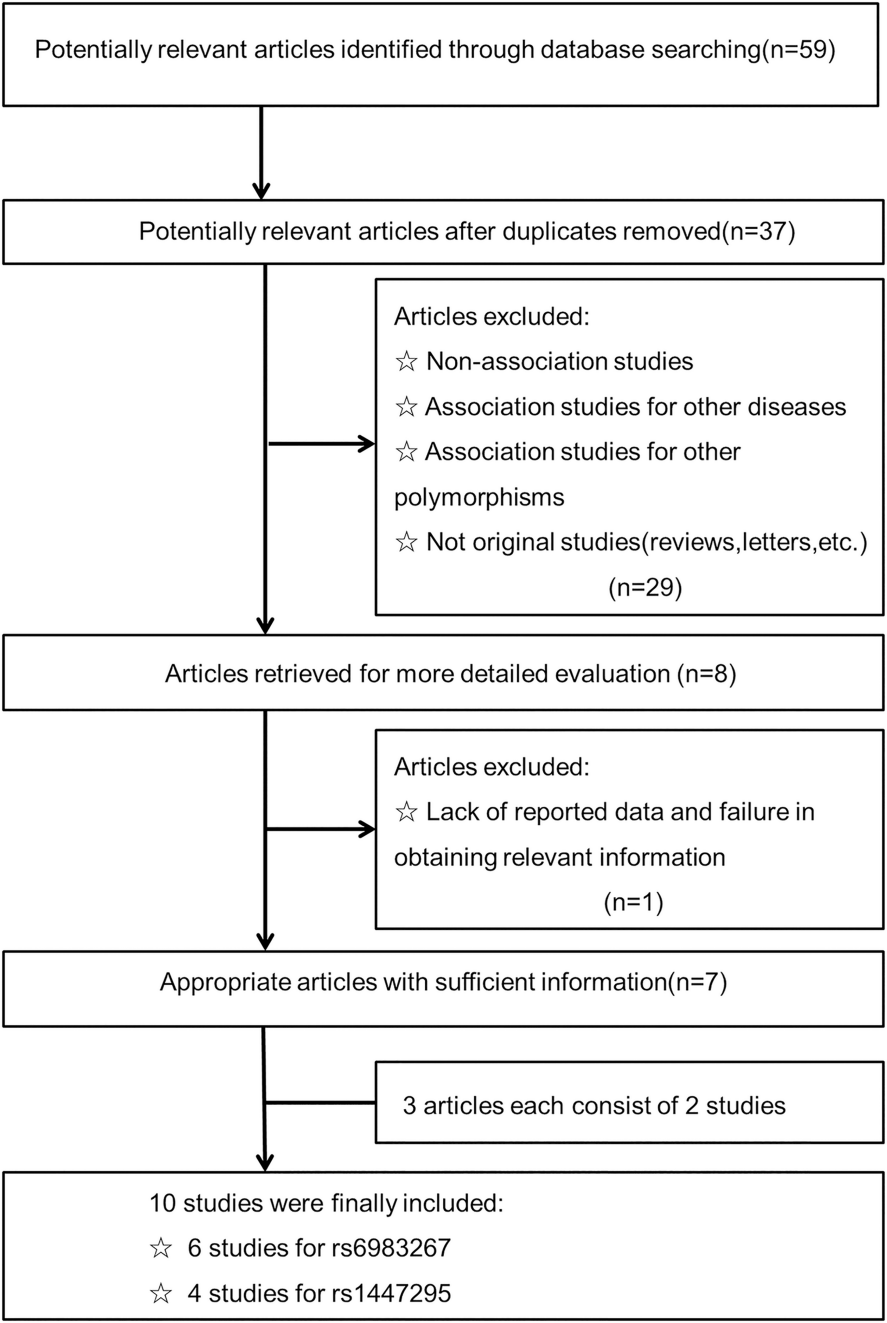
Flow diagram of the selection of eligible studies.

A total of 59 potentially relevant publications were retrieved based on our enrollment selection criteria; 37 full-text references were finally identified after removal of duplicates (Fig 1). After a further careful analysis of the remaining study titles and abstracts, 29 were excluded. Of the remaining eight eligible studies, one did not have enough available data and was subsequently excluded due to failure to obtain relevant information from the corresponding authors [24]. A manual search of references cited in the eligible articles did not reveal any additional studies. Consequently, seven articles including ten case-control studies met our inclusion criteria, of these six studies investigated rs6983267 polymorphisms and GC susceptibility had a total of 4,813 subjects [12, 13, 15–18] and four studies investigated rs1447295 variants in 2,054 subjects [14, 16–18]. Detailed characteristics and genotype distribution of eligible studies are summarized in Tables 1 and 2, respectively. Of all the eligible studies, six were conducted in an Asian population, two in an European population and two in a South American population. The mean age was documented in four studies, ranging from 58 to 63 years of age. The ratio of *H*. *pylori* infection was described in only one article. Five studies used either a restriction fragment length polymorphism polymerase chain reaction (RFLP-PCR) or TaqMan assay to genotype any polymorphisms. The genotype distribution of the controls in three studies was not in compliance with the Hardy-Weinberg equilibrium (HWE); however, our quality assessment ensured all studies were of high quality with a NOS scores of more than five stars.

**Table 1:** Characteristics of studies included in the meta-analysis.

Variant	Study	Year	Country	Ethnicity	Sample size (men%)		Mean age (year)		H. pylori infection (%)		source of control	Genotyping method	NOS
					case	control	case	control	Case	control			
**rs1447295**	Lochhead P[14]	2011	Poland	European	286	365	-	-	-	-	population based	RT-PCR	8
	Labrador L[18]	2015a	Venezuela	South American	122	129	-	-	-	-	population based	RFLP-PCR	6
	Park SL[16]	2008a	China	Asian	187	388	-	-	-	-	population based	TaqMan	8
	Tarleton HP[17]	2014a	china	Asian	184(75)	384(74.7)	61.5	57.7	71(38.6)	114(29.7)	population based	TaqMan	8
**rs6983267**	Guo Y[12]	2011	China	Asian	200(76)	368(65.8)	62.47	62.89	-	-	population based	RFLP-PCR	8
	Park SL[16]	2008b	China	Asian	187	385	-	-	-	-	population based	TaqMan	8
	Labrador L[18]	2015b	Venezuela	South American	122	129	-	-	-	-	population based	RFLP-PCR	6
	Tarleton HP[17]	2014b	china	Asian	178(77.5)	374(76.6)	61.5	57.7	71(39.9)	114(30.5)	population based	TaqMan	8
	Wokolorczyk D[15]	2008	Poland	European	488	1910(46.6)	58.5	61.1	-	-	population based	RFLP-PCR	8
	Zhou CP[13]	2014	china	Asian	246(71.5)	227(58.1)	-	-	-	-	population based	MassARRAY	8

NOS: Newcastle-Ottawa Scale; *H*. *pylori*: *helicobacter pylori*.

**Table 2:** Genotype distribution among studies included in the meta-analysis.

Study	Year	Country	rs6983267 case/control				rs1447295 case/control			
			TT	GT	GG	HWE	CC	CA	AA	HWE
Wokolorczyk D	2008	Poland	126/513	255/977	107/420	Yes	-	-	-	-
Park SL	2008a	China	-	-	-	-	140/276	39/101	8/11	Yes
Park SL	2008b	China	61/146	94/165	32/74	No	-	-	-	-
Guo Y	2011	China	32/72	134/196	34/100	Yes	-	-	-	-
Lochhead P	2011	Poland	-	-	-	-	244/287	39/67	3/11	Yes
Tarleton HP	2014a	China	-	-	-	-	140/275	37/100	7/9	No
Tarleton HP	2014b	China	58/143	89/160	31/71	No	-	-	-	-
Zhou CP	2014	China	70/74	125/106	51/47	Yes	-	-	-	-
Labrador L	2015a	Venezuela	-	-	-	-	76/86	35/35	11/8	Yes
Labrador L	2015b	Venezuela	26/27	47/56	49/46	Yes	-	-	-	-

HWE, Hardy-Weinberg equilibrium.

### Meta-analysis of rs6983267 polymorphisms

**Fig 2:**
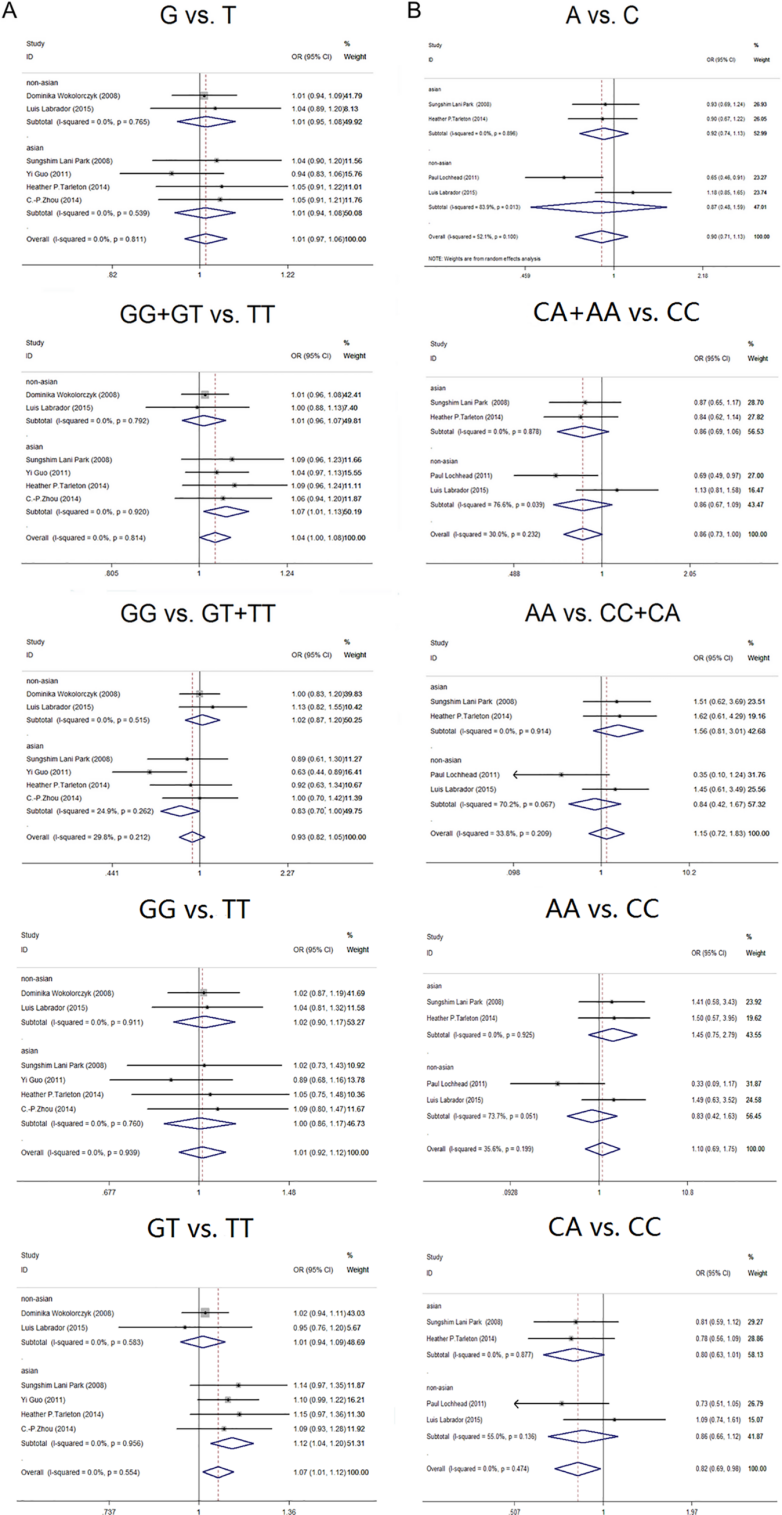
Forrest plots for the relationship between variants at 8q24 and gastric cancer under allele, dominant, recessive homozygote or heterozygote models. A. For rs6983267; B. For rs1447295. The solid squares represent odds ratios (OR) from individual studies; the diamonds are shown as overall effect.

Six studies included a total of 1,421 GC patients and 3,393 control cases genotypically examining rs6983267 SNP (Table 3). As Fig 2A shows, the pooled results indicated that there is significant association between the rs6983267 SNP and GC susceptibility utilizing a heterozygote model (GT vs. TT: OR = 1.07, 95% CI = 1.01–1.12, *P* = 0.015). In further sub-analysis based on ethnicity and genotyping method, similar results were identified taking into account differences in Asian population and other genotyping method subgroup (OR = 1.12, 95% CI = 1.04–1.20, *P* = 0.003; OR = 1.13, 95% CI = 1.02–1.14, *P* = 0.015; respectively). Although there was no significant association between the rs6983267 SNP and GC susceptibility in dominant model (GG+GT vs. TT: OR = 1.04, 95% CI = 1.00–1.08, *P* = 0.047), GG+GT genotype carriers suffered 18% elevated risk of developing gastric cancer than TT carriers in Asian subgroup (32.1% and 27.3%, respectively, OR = 1.07, 95% CI = 1.01–1.13; *P* = 0.02). However, after making a Bonferroni correction for multiple testing, the association was observed to be non-significant. No evidence for an association was observed using the other three genetic models (G vs. T: OR = 1.01, 95% CI = 0.97–1.06, *P* = 0.588; GG vs. GT+TT: OR = 0.93, 95% CI = 0.82–1.05, *P* = 0.236; GG vs. TT: OR = 1.01, 95% CI = 0.92–1.12, *P* = 0.780). Overall, there was no significant inter-study heterogeneity using the allele, dominant, recessive, homozygote, heterozygote models (*I*^*2*^ were 0%, 0%, 29.8%, 0% and 0%, respectively; see Table 3 and Table 4). However, obvious heterogeneity was seen in recessive models using a RFLP-PCR genotyping method (*I*^*2*^ = 71.1%). Sensitivity analysis presented numerical similarity and the same conclusion after deleting each methodone by one (S1–S5 Figs). Publication bias was not statistically significant among the enrolled studies as shown in Table 5 (Egger’s test: G vs. T: *P* = 0.536; GG+GT vs. TT: *P* = 0.202; GG vs. GT+TT: *P* = 0.484, GG vs. TT: *P* = 0.947, GT vs. TT: *P* = 0.550).

**Table 3:** Meta-analysis of rs6983267 and gastric cancer.

	Studies(n)	Sample size Case/control	G vs. T				GG+GT vs. TT				GG vs. GT+TT			
			OR(95%CI)	*P*	*I*_*2*_ (%)	*P*_*Q*_	OR(95%CI)	*P*	*I*_*2*_ (%)	*P*_*Q*_	OR(95%CI)	*P*	*I*_*2*_ (%)	*P*_*Q*_
**overall**	6	1421/3393	1.01 [0.97,1.06]	0.588	0.0	0.811	1.04 [1.00,1.08]	0.047	0.0	0.814	0.93 [0.82,1.05]	0.236	29.8	0.212
**Ethnicity**														
Non-Asian	2	610/2039	1.01 [0.95,1.08]	0.670	0.0	0.765	1.01 [0.96,1.07]	0.680	0.0	0.792	1.02[0.87,1.20]	0.775	0.0	0.515
Asian	4	811/1354	1.01 [0.94,1.08]	0.734	0.0	0.539	***1*.*07 [1*.*01*,*1*.*13]***	0.020	0.0	0.920	0,83 [0.70,1.00]	0.050	24.9	0.262
**Genotyping method**														
PCR-RFLP	3	810/2407	1.00 [0.94,1.06]	0.898	0.0	0.499	1.02[0.97,1.07]	0.407	0.0	0.768	0.91 [0.67,1.22]	0.515	71.1	0.031
other	3	611/986	1.05 [0.96,1.14]	0.297	0.0	0.995	1.08[1.00,1.16]	0.039	0.0	0.946	0.94 [0.76,1.16]	0.558	0.0	0.896

**Table 4:** Meta-analysis of rs6983267 and gastric cancer.

	Studies(n)	Sample size Case/control	GG vs. TT				GT vs. TT			
			OR(95%CI)	*P*	*I*_*2*_ (%)	*P*_*Q*_	OR(95%CI)	*P*	*I*_*2*_ (%)	*P*_*Q*_
**overall**	6	1421/3393	1.01 [0.92,1.12]	0.780	0.0	0.939	**1.07 [1.01,1.12]**	0.015	0.0	0.554
**Ethnicity**										
Non-Asian	2	610/2039	1.02 [0.90,1.17]	0.729	0.0	0.911	1.01 [0.94,1.09]	0.736	0.0	0.583
Asian	4	811/1354	1.00 [0.86,1.17]	0.959	0.0	0.760	**1.12 [1.04,1.20]**	0.003	0.0	0.956
**Genotyping method**										
PCR-RFLP	3	810/2407	1.02 [0.88,1.12]	0.940	0.0	0.625	1.04 [0.97,1.10]	0.267	3.5	0.355
other	3	611/986	1.05 [0.87,1.27]	0.589	0.0	0.968	**1.13 [1.02,1.24]**	0.015	0.0	0.884

**Table 5:** Publication bias test.

		rs6983267					rs1447295				
		G vs. T	GG+GT vs. TT	GG vs. GT+TT	GG vs. TT	GT vs. TT	A vs. C	CA+AA vs. CC	AA vs. CC+CA	AA vs. CC	CA vs.CC
**Egger’ Test**	***P* value**	0.536	0.202	0.484	0.947	0.550	0.784	0.963	0.066	0.052	0.363
	**95% CI**	[-2.12, 3.48]	[-0.94, 3.23]	[-6.39, 3.61]	[-2.12, 2,.23]	[-2.37, 3.82]	[-62.2,53.7]	[-48.82,47.64]	[-15.73,1.16]	[-14.75,0.16]	[-18.32,31.96]

### Meta-analysis of rs1447295 polymorphisms

Four studies included a total of 779 GC patients and 1,266 control cases undergoing rs1447295 genotypic analysis (Table 6 and Table 7). In this case there was a significant reduction of GC susceptibility in CA carriers compared with CC carriers under a heterozygote model (CA vs. CC: OR = 0.82, 95% CI = 0.69–0.98, *P* = 0.030). However, Bonferroni correction for multiple testing, the association was found to be non-significant. There was no significant association between rs1447295 and gastric cancer susceptibility using an allele, dominant, recessive, homozygote or heterozygote genetic models (A vs. C: OR = 0.90, 95% CI = 0.71–1.13, *P* = 0.350; CA+AA vs. CC: OR = 0.86, 95% CI = 0.73–1.00, *P* = 0.054; AA vs. CC+CA: OR = 1.15, 95% CI = 0.72–1.83, *P* = 0.595; AA vs. CC: OR = 1.10, 95%CI = 0.69–1.76, *P* = 0.681; Fig 2B). In subgroup analyses, similar results were identified taking into account differences in ethnicity and genotyping methods. Significant inter-study heterogeneity was observed when the allele model was used (*I*^*2*^ = 52.1%, Table 6). In subgroup analyses, this heterogeneity was not seen in the Asian population as well as in the subgroup using a TaqMan genotyping method. Our sensitivity analysis suggested that there was no significant change in the overall outcomes after removing any of the studies (S6–S10 Figs). An Egger’s test were done in all the three models showing that there was no statistical evidence for publication bias except in the recessive and homozygote models (A vs. C: *P* = 0.784; CA+AA vs. CC: *P* = 0.963; AA vs. CC+CA: *P* = 0.066; AA vs. CC: *P* = 0.052; CA vs. CC:*P* = 0.363; Table 5).

**Table 6:** Meta-analysis of rs1447295 and gastric cancer.

	Studies(n)	Sample size Case/control	A vs. C				CA+AA vs. CC				AA vs. CC+CA			
			OR(95%CI)	*P*	*I*_*2*_ (%)	*P*_*Q*_	OR(95%CI)	*P*	*I*_*2*_ (%)	*P*_*Q*_	OR(95%CI)	*P*	*I*_*2*_ (%)	*P*_*Q*_
**overall**	4	779/1266	0.90 [0.71,1.13]	0.350	52.1	0.100	0.86 [0.73,1.00]	0.054	30.0	0.232	1.15 [0.72,1.83]	0.595	33.8	0.209
**Ethnicity**														
Non-Asian	2	408/672	0.87 [0.48,1.59]	0.658	83.9	0.013	0.88 [0.54,1.45]	0.621	76.6	0.039	0.77 [0.19,3.12]	0.185	70.2	0.067
Asian	2	371/494	0.92 [0.74,1.13]	0.411	0.0	0.896	0.86 [0.69,1.06]	0.150	0.0	0.878	1.56 [0.81,3.01]	0.711	0.0	0.914
**Genotyping method**														
TaqMan	2	371/494	0.92 [0.74,1.13]	0.411	0.0	0.896	0.86 [0.69,1.06]	0.150	0.0	0.878	1.56 [0.81,3.01]	0.711	0.0	0.914
other	2	408/672	0.87 [0.48,1.59]	0.658	83.9	0.013	0.88 [0.54,1.45]	0.621	76.6	0.039	0.77 [0.19,3.12]	0.185	70.2	0.067

**Table 7:** Meta-analysis of rs1447295 and gastric cancer.

	Studies(n)	Sample size Case/control	AA vs. CC				CA vs. CC			
			OR(95%CI)	*P*	*I*_*2*_ (%)	*P*_*Q*_	OR(95%CI)	*P*	*I*_*2*_ (%)	*P*_*Q*_
**overall**	4	779/1266	1.10 [0.69,1.76]	0.681	35.6	0.199	**0.82 [0.69,0.98]**	0.030	0.0	0.474
**Ethnicity**										
Non-Asian	2	408/672	0.83 [0.43,1.63]	0.594	73.7	0.051	0.86 [0.66,1.12]	0.261	55	0.136
Asian	2	371/494	1.45 [0.76,2.79]	0.264	0.0	0.925	0.80 [0.63,1.01]	0.057	0.0	0.887
**Genotyping method**										
TaqMan	2	371/494	1.45 [0.76,2.79]	0.264	0.0	0.925	0.80 [0.63,1.01]	0.057	0.0	0.887
other	2	408/672	0.83 [0.43,1.63]	0.594	73.7	0.051	0.86 [0.66,1.12]	0.261	55	0.136

## Discussion

Thus far, hundreds of studies have linked specific gene polymorphisms to the risk of developing gastric carcinoma, and among those several high-quality biomarkers those with gastric cancer susceptibility associations have been identified [25]. According to our calculations, “gene deserts” excluded in these types of analyses leave an estimated 25% of the entire human genome uninvestigated [26]. Recent genome-wide association studies (GWAS) routinely identify risk variations within “gene deserts” and other types of noncoding DNA in the etiology of various cancers. On the 8q24 chromosome, there is 1.5 Mb “gene desert” bounded by the gene *FAM84B* on the centromeric end and the proto-oncogene *c-MYC* on the telomeric end. Within this gene desert there are SNPs reported to be associated with multiple types of cancers, including: breast [9], prostate [11], colorectal [10], and urinary bladder [27]. Recently, a growing number of epidemiological studies have been performed to evaluate the relationship between 8q24 polymorphisms (specifically rs6983267 and rs1447295) and GC susceptibility. Guo *et al*. first reported that a SNP in rs6983267 increased the susceptibility to GC in a Chinese population [12]. Soon afterwards another research group came to the same conclusion using a Chinese female subgroup [13]. At the same time, Lochhead *et al*. found a novel inverse association between rs1447295 SNP and GC susceptibility [14]. However, other studies have declared a negative association between 8q24 (rs6983267 and rs1447295) polymorphisms and GC susceptibility [15–18]. Therefore, we carried out a meta-analysis of cancer biology field including case-control research to make a more accurate assessment of the relationship between these SNPs and gastric cancer susceptibility.

In our analysis, six studies of the rs6983267 variant were included, with a total of 1,421 GC patients and 3,393 control subjects. We then assessed the association between 8q24 rs6983267 and GC susceptibility. Our pooled results showe a significant association between the rs6983267 polymorphism and GC susceptibility using a heterozygote genetic model. When we conducted a sub-analysis of rs6983267 based on ethnicity, we found that GG+GT genotype carriers may be 18% more susceptible to gastric cancer than TT carriers in Asian population. To achieve a more reliable result, we used a Bonferroni correction to compensate for multiple testing. After this correction both significant associations between rs6983267 variant and GC were no longer observed indicating that further well-designed prospective large-scale studies are warranted.

To our knowledge, multiple gastric cancer–associated sequence variants at 8q24 could affect the expression of some genes in the regions that flank these variants (the *cis* effect) as well as genes in other regions of the genome (the *trans* effect). Among those genes the proto-oncogene *c-MYC*, located 330 kb away from SNP rs6983267, has been implicated in human cancers by influencing several important cellular functions including: the cell cycle, apoptosis, signal transduction, transcriptional and post-transcriptional regulatory mechanisms, non-coding RNAs, stem cell biology, chromosomal translocation and gene amplification [28]. In human gastrointestinal carcinoma, defects in the Wnt-APC pathway results in enhanced T-cell factor 4 (TCF4) transcriptional activation of *c-MYC* [29]. In this pathway, TCF4, also called TCF7L2, forms a complex with beta-catenin, and the beta-catenin–TCF4 transcription factor complex binds preferentially to the cancer risk-associated rs6983267 (G) allele *in vivo* and *in vitro* leading to a two-fold change in *c-MYC* expression [30]. In addition to proto-oncogene *c-MYC*, pseudogene *POU5F1P1* resides only 15 kb away from SNP rs6983267. It is known that *POU5F1P1* is remarkably homologous to *OCT4A* (95% homology), which is considered to be a key regulator of pluripotency as well as for the propagation of mammalian germlines [31, 32]. Moreover, recent research has reported that the pseudogene *POU5F1P1* is amplified and expressed at a high level in gastric cancer and confers an aggressive phenotype, leading to a poor prognosis in subjects with GC [33].

In our rs1447295 variant meta-analysis, four studies were adopted including 779 GC patients and 1,266 control cases to evaluate the relationship between SNP rs1447295 and GC susceptibility. This variant is previously implicated in prostate and colorectal cancer susceptibility, and our study finds it maybe a protective factor in terms of gastric cancer utilizing the heterozygote genetic model. The function of this variant may play a different role in distinct cancers. After a multiple testing correction to our calculations, the association was not significant hinting that a large scale association study may be need to be conducted.

Several limitations of our analyses need to be taken into consideration. First, we made meticulous efforts to obtain relevant articles only through electronic databases, leading to a potential bias caused by an absence of unpublished data and the exclusion of one study [14] which could not be included due to a lack of data. Second, our study is a summary of the relevant data. Due to the lack of the individual raw data, we could not evaluate gastric cancer susceptibility stratified by other variables such as age, gender distribution, *H*. *pylori* infection, alcohol consumption, cigarette smoking and other risk factors. Third, we were limited in our ability to analyze all gene-to-environment and gene-to-gene interactions. Finally, our meta-analysis included several studies with small sample sizes, which may influence the reliability of our results.

In conclusion, there was no evidence to support the hypothesis that the rs6983267 and rs1447295 variants had any influence on the susceptibility of GC in this meta-analysis. Further haplotype research and haplotypic meta-analyses should be preferentially conducted to provide more powerful and informative conclusions about gastric cancer susceptibility than currently published SNP studies.

## Supporting information

S1 Table The full electronic search strategy for Pubmed. (DOCX)

S1 Fig Sensitivity analysis test for the association between gastric cancer and rs6983267 under allele model. (TIF)

S2 Fig Sensitivity analysis test for the association between gastric cancer and rs6983267 under dominant model. (TIF)

S3 Fig Sensitivity analysis test for the association between gastric cancer and rs6983267 under recessive model. (TIF)

S4 Fig Sensitivity analysis test for the association between gastric cancer and rs6983267 under homozygote model. (TIF)

S5 Fig Sensitivity analysis test for the association between gastric cancer and rs6983267 under heterozygote model. (TIF)

S6 Fig Sensitivity analysis test for the association between gastric cancer and rs1447295 under allele model. (TIF)

S7 Fig Sensitivity analysis test for the association between gastric cancer and rs1447295 under dominant model. (TIF)

S8 Fig Sensitivity analysis test for the association between gastric cancer and rs1447295 under recessive model. (TIF)

S9 Fig Sensitivity analysis test for the association between gastric cancer and rs1447295 under homozygote model. (TIF)

S10 Fig Sensitivity analysis test for the association between gastric cancer and rs1447295 under heterozygote model. (TIF)

S1 File Plosone-meta-analysis-checklist. (DOCX)

S2 File PRISMA 2009 checklist. (DOC)

S3 File Database 1. (MYD)

S4 File Database 2. (FRM)

## Abbreviations

GC: gastric cancer
SNP: single nucleotide polymorphism
OR: Odds ratio
CI: Confidence interval
*H. pylori*: *helicobacter pylori*
NOS: Newcastle-Ottawa Scale
RFLP-PCR: polymerase chain reaction-restriction fragment length polymorphism
HWE: Hardy-Weinberg equilibrium
TCF4: T-cell factor 4
