# Supplementary material for: Genetic variations at 8q24 and gastric cancer susceptibility: A meta-analysis study
Source: PLoS One. 2017 Dec 12;12(12):e0188774. doi: 10.1371/journal.pone.0188774 (PMC5726661; doi:10.1371/journal.pone.0188774)
Supplement: S1 Table — (DOCX) [file pone.0188774.s001.docx]

Table 1. The full electronic search strategy for Pubmed database

| Search | Query | Items found |
| --- | --- | --- |
| #4 | **((#1) AND #2) AND #3** | 11 |
| #3 | **((8q24[Title/Abstract]) OR rs1447295[Title/Abstract]) OR rs6983267[Title/Abstract]** | 1721 |
| #2 | **(((polymorphism[Title/Abstract]) OR variation[Title/Abstract]) OR allele[Title/Abstract]) OR genotype[Title/Abstract]** | 664426 |
| #1 | Search ((((((((gastric cancer[Title/Abstract]) OR gastric carcinoma[Title/Abstract]) OR gastric tumor[Title/Abstract]) OR gastric neoplasms[Title/Abstract]) OR stomach cancer[Title/Abstract]) OR stomach carcinoma[Title/Abstract]) OR stomach tumor[Title/Abstract]) OR stomach neoplasms[Title/Abstract]) | 64636 |
