# Supplementary figures and images for: Genetic variations at 8q24 and gastric cancer susceptibility: A meta-analysis study
Source: PLoS One. 2017 Dec 12;12(12):e0188774. doi: 10.1371/journal.pone.0188774 (PMC5726661; doi:10.1371/journal.pone.0188774)

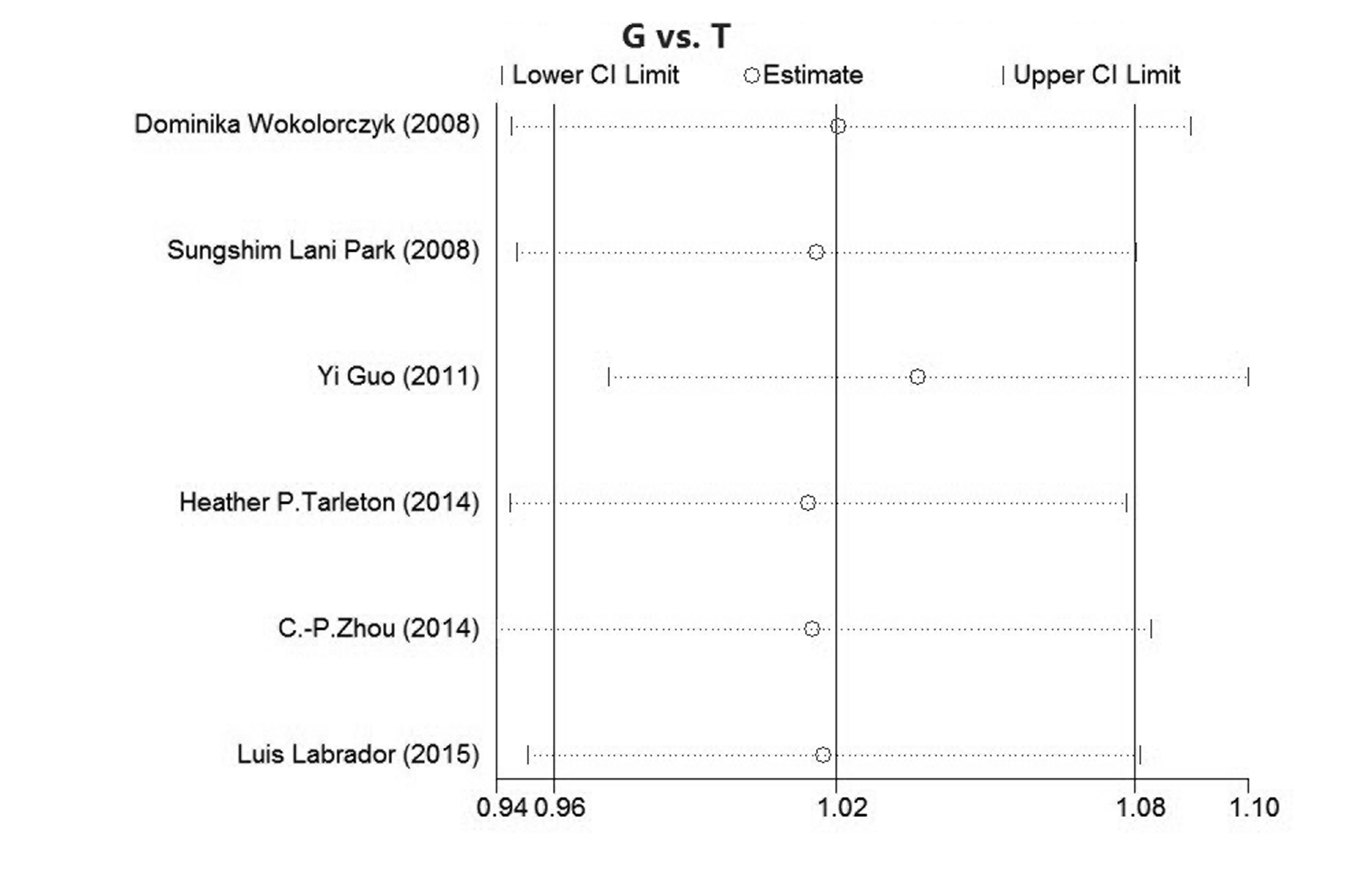

Supplement: S1 Fig — (TIF) [file pone.0188774.s002.tif]

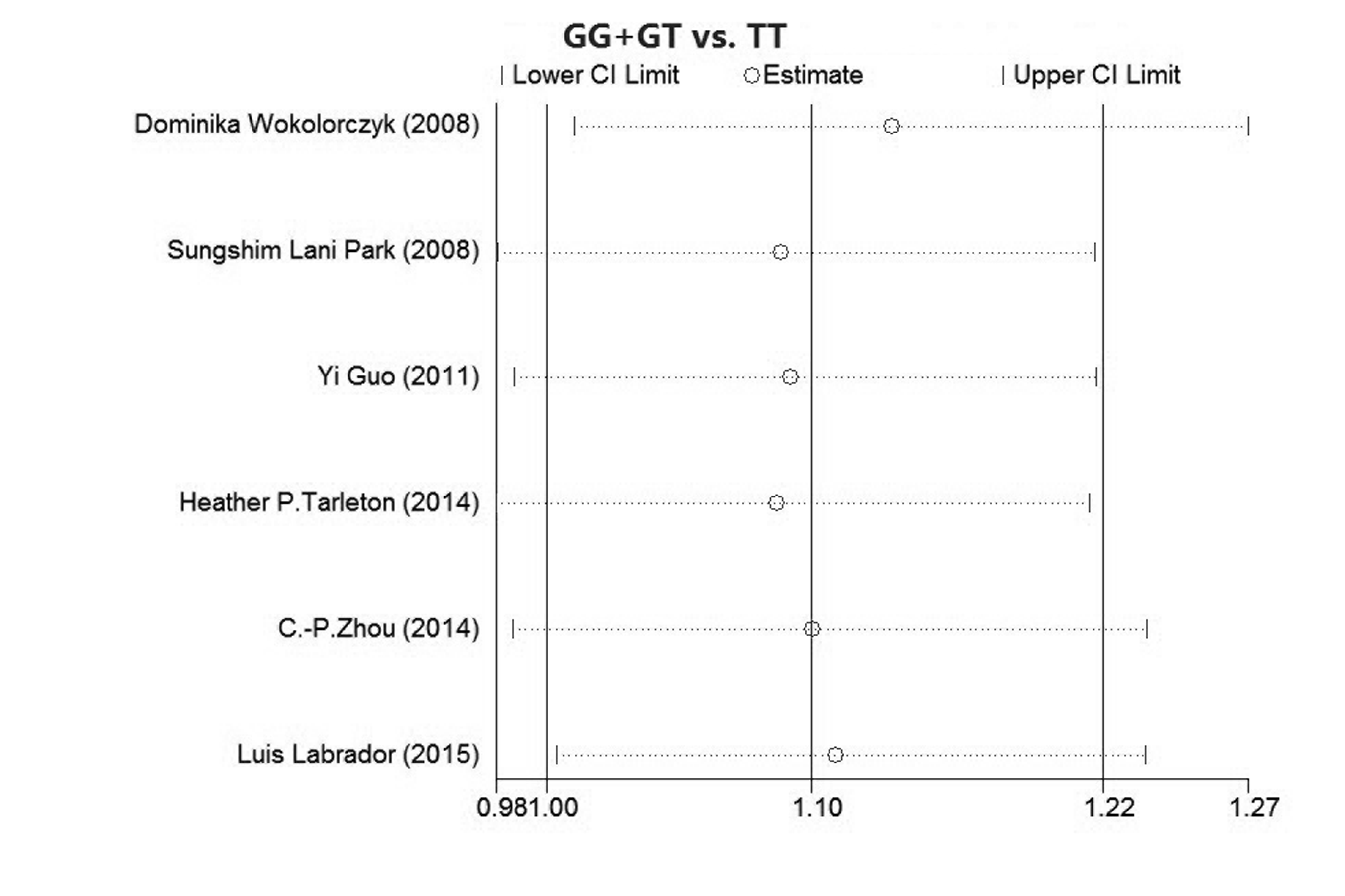

Supplement: S2 Fig — (TIF) [file pone.0188774.s003.tif]

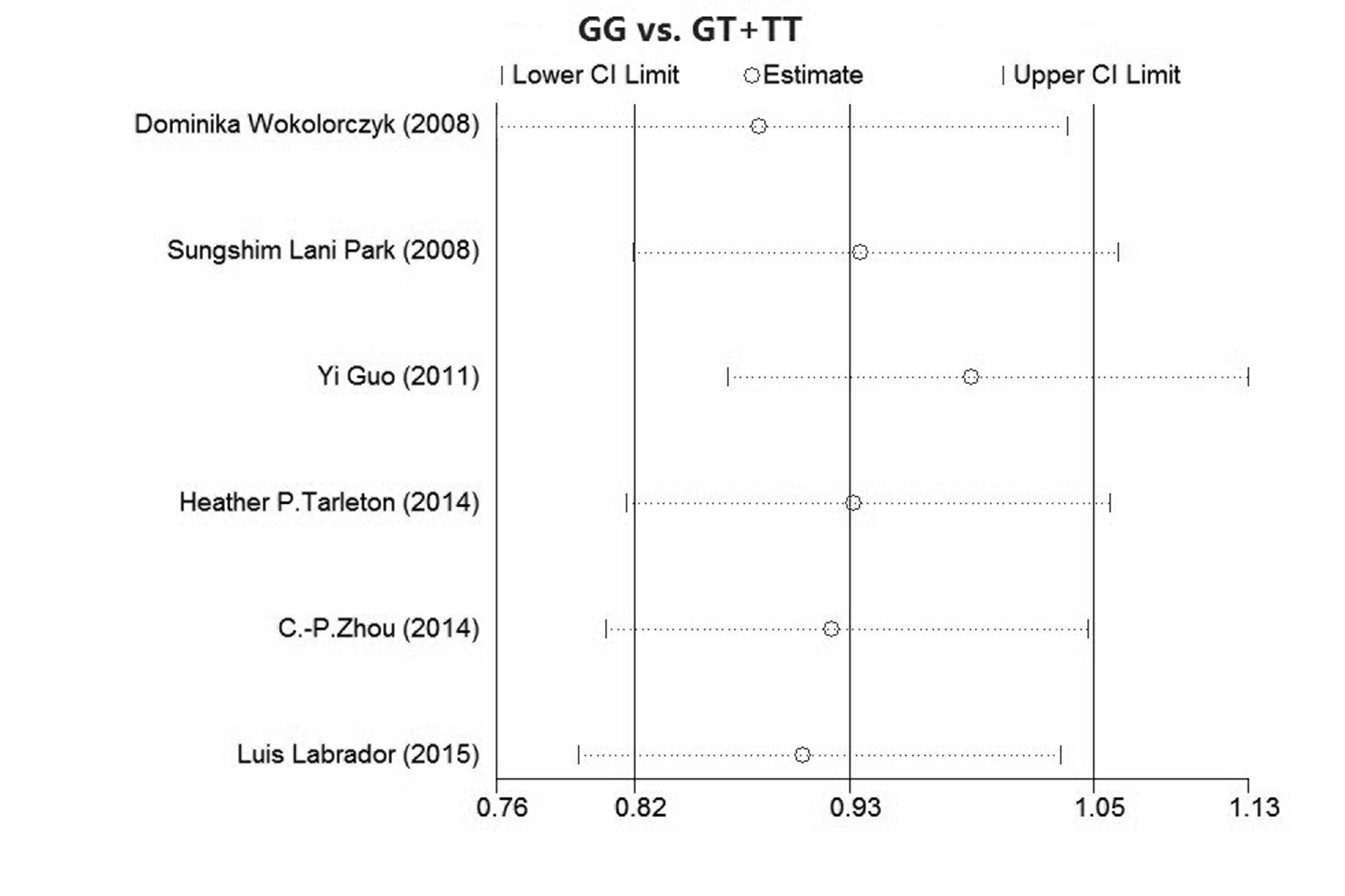

Supplement: S3 Fig — (TIF) [file pone.0188774.s004.tif]

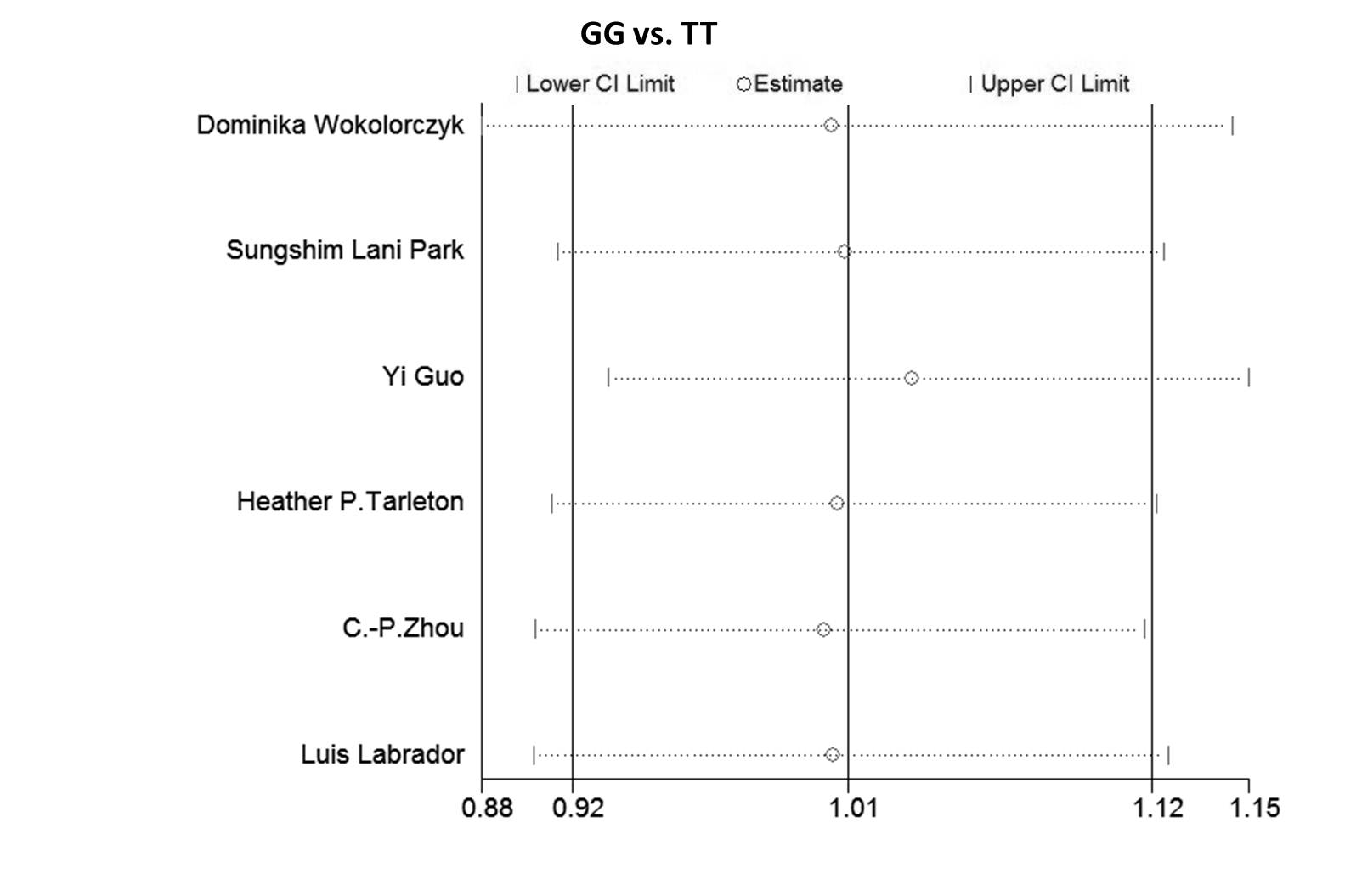

Supplement: S4 Fig — (TIF) [file pone.0188774.s005.tif]

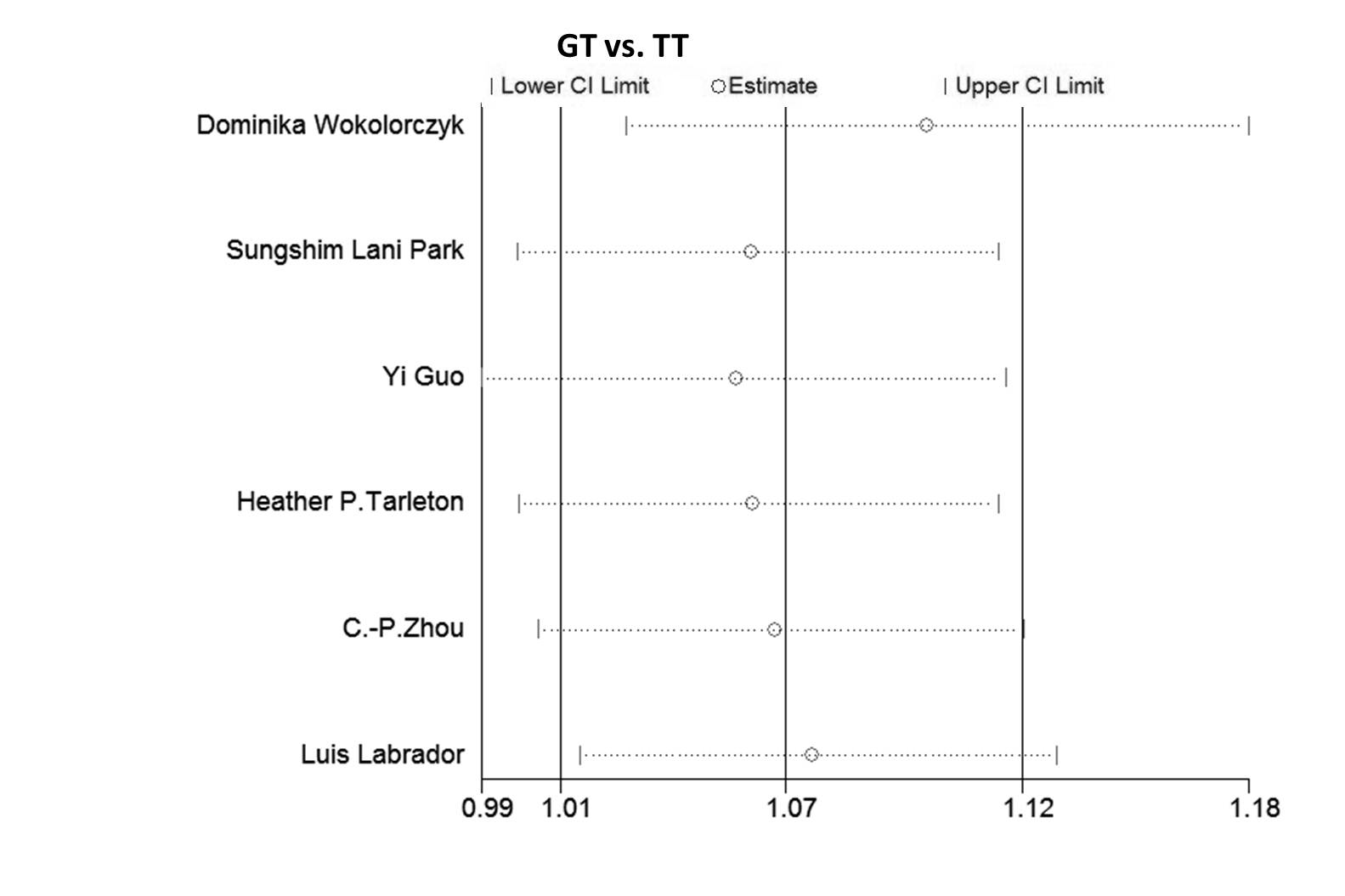

Supplement: S5 Fig — (TIF) [file pone.0188774.s006.tif]

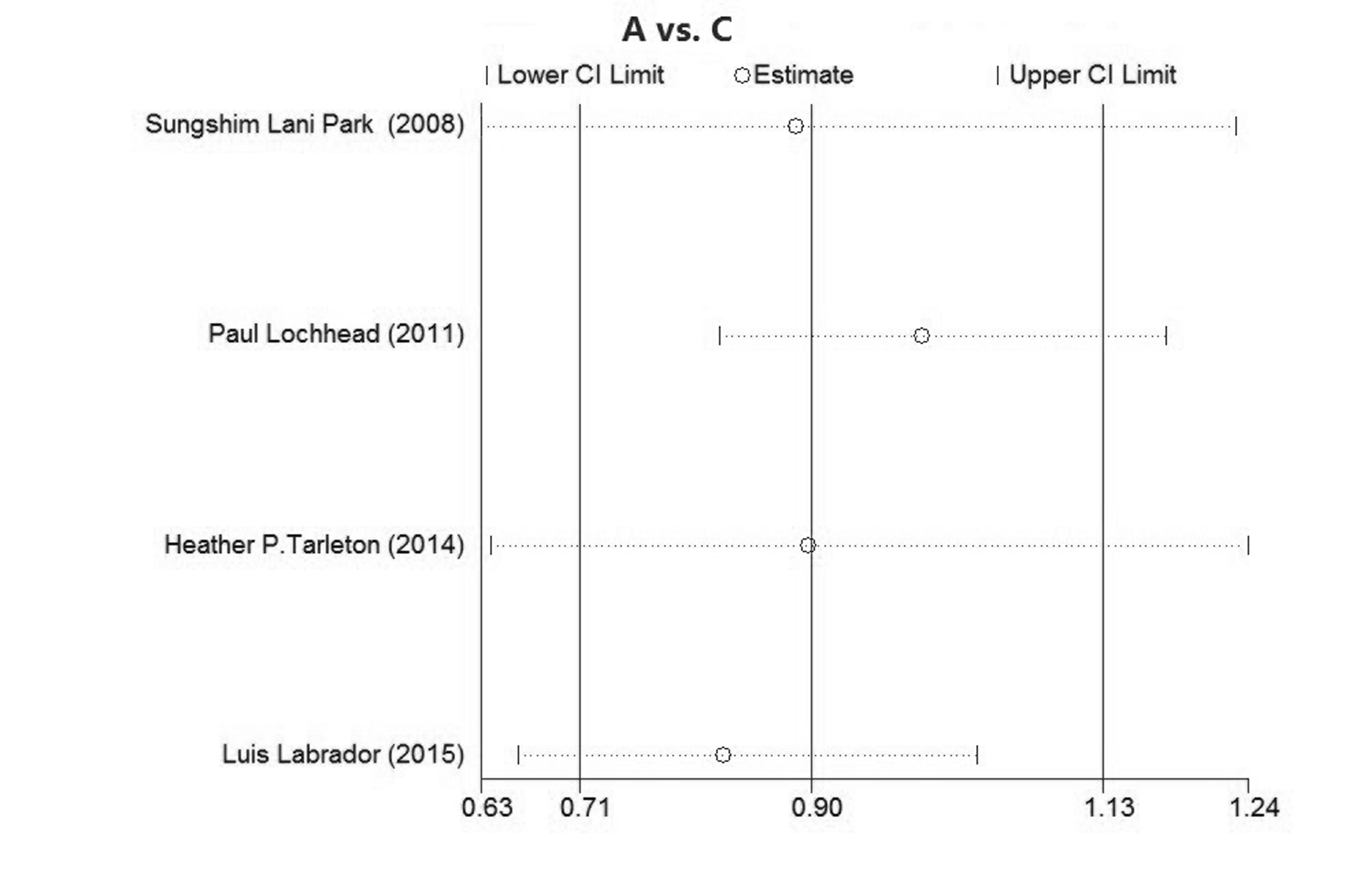

Supplement: S6 Fig — (TIF) [file pone.0188774.s007.tif]

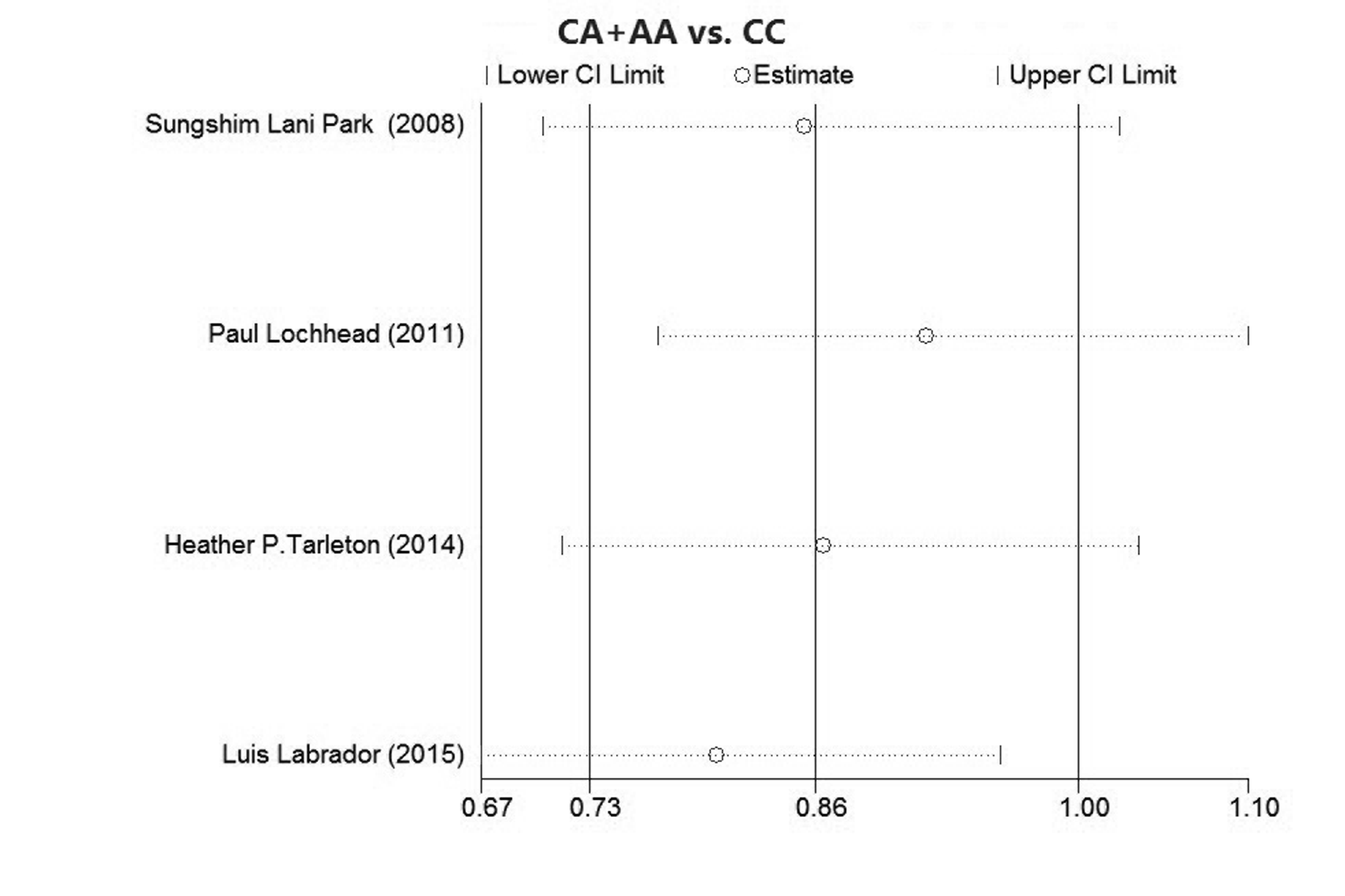

Supplement: S7 Fig — (TIF) [file pone.0188774.s008.tif]

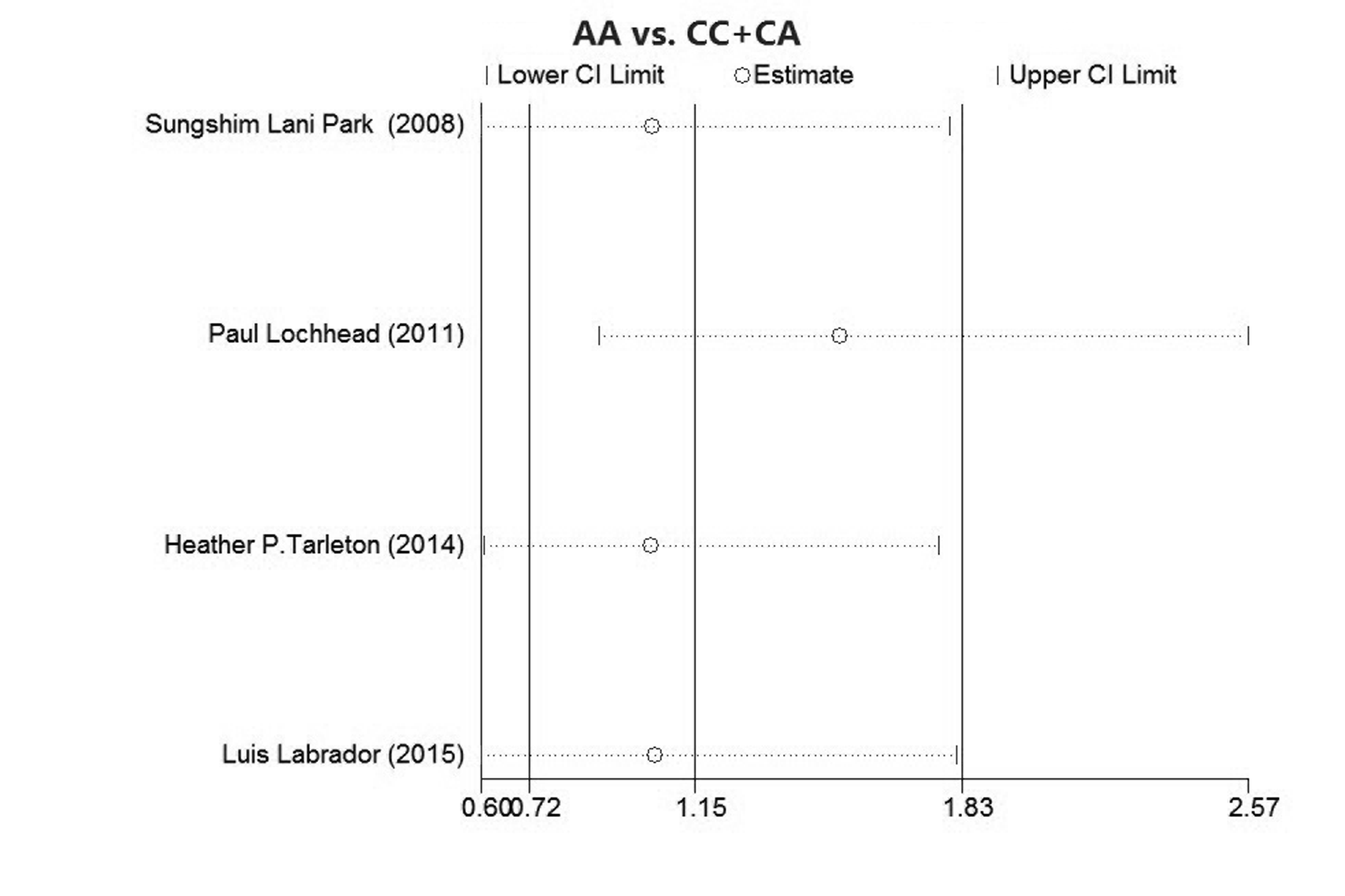

Supplement: S8 Fig — (TIF) [file pone.0188774.s009.tif]

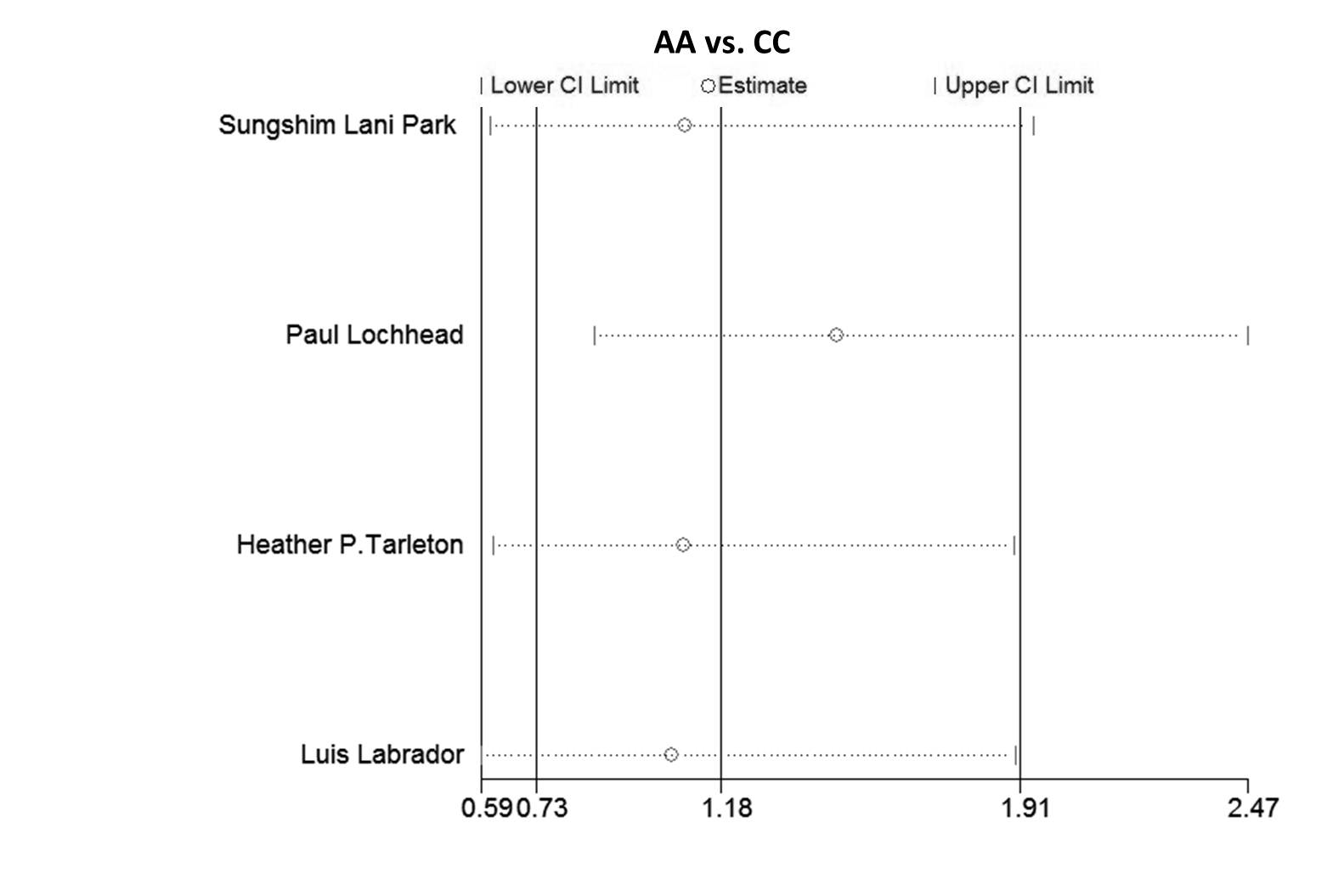

Supplement: S9 Fig — (TIF) [file pone.0188774.s010.tif]

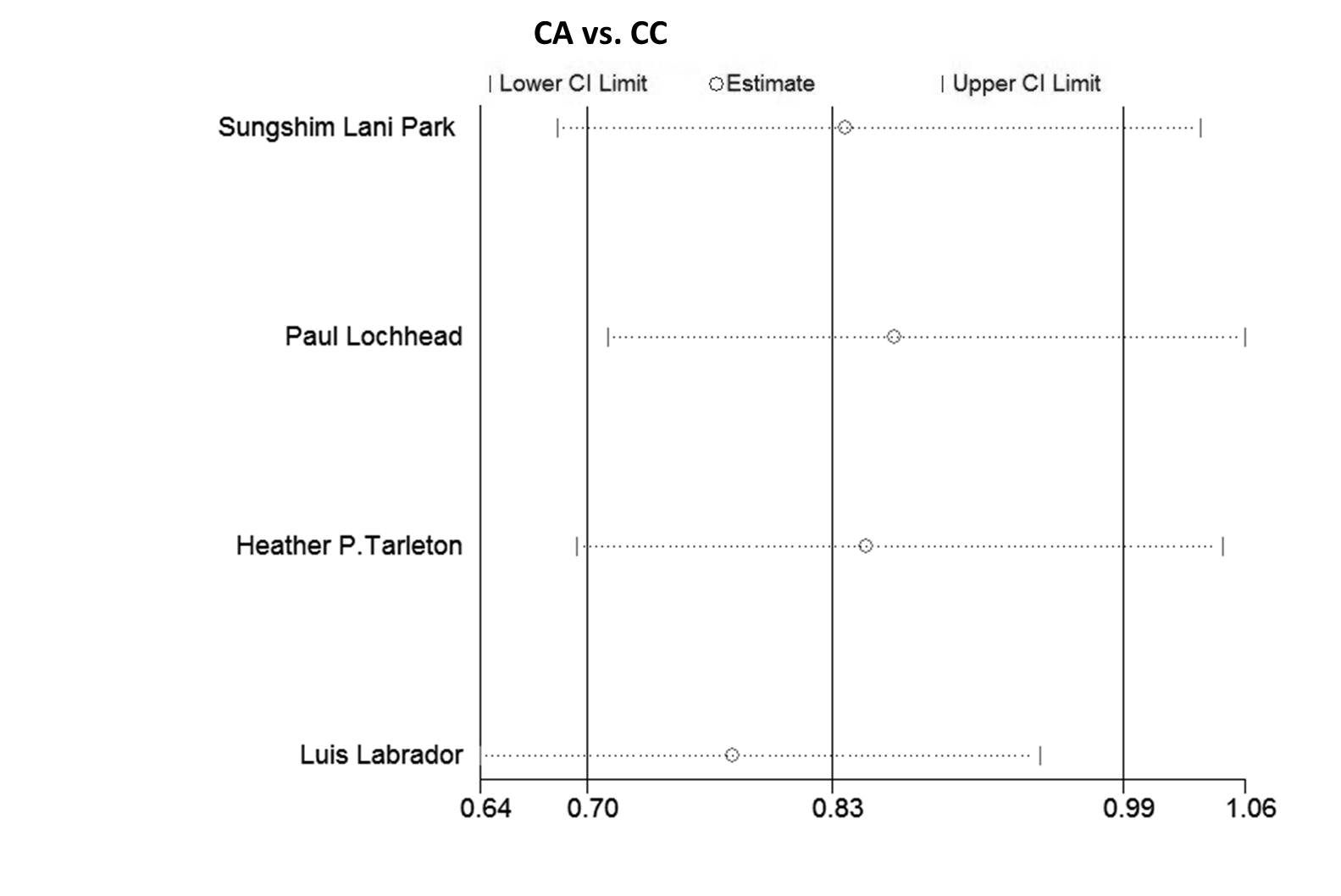

Supplement: S10 Fig — (TIF) [file pone.0188774.s011.tif]
